# Assessment of impulsivity and other psychological factors in obese patients

**DOI:** 10.1192/j.eurpsy.2022.1500

**Published:** 2022-09-01

**Authors:** I. Martin Villalba

**Affiliations:** Hospital Clinic de Barcelona, Psychiatry And Clinical Psychology, Barcelona, Spain

**Keywords:** Impulsivity, obesity

## Abstract

**Introduction:**

Obesity is a multiorgan disorder that is caused by eating a much larger overeating that the body needs to live. Obese people tend to eat a lot and after hours, and it is hypothetised that it exists a relationship between increased impulsivity and obesity. In addition these patients tend to have more psychopathology,

**Objectives:**

The aim of the study is to observe if obese people ingest impulsively and if there are differences between the sexes regarding impulsivity. Also it will be studied the relationship between impulsivity in these patients and other psychosocial factors, anxiety and depression symptoms and personality traits.

**Methods:**

It was carried out an assessment of impulsivity in 30 obese population followed at the outpatient Endocrinology that attended pre-bariatric surgery groups.Patients were handed the Plutchik impulsivity questionnaire, Hospital Anxiety and Depression Scale (HADS) and The Temperament and Character Inventory (TCI).

**Results:**

In our sample, 43.4% of the obese patients did not show high levels of impulsivity, while 56.6% did show markedly impulsive traits. No differences in impulsivity between sexes were found. No other significant relationships with addititional psychological factors were found.

**Conclusions:**

A larger sample is needed to reach a conclusion and to extrapolate the results to the general population. People with morbid obesity have higher impulsivity and a binge eating;in addition they have more psychopathology, mainly affective, greater impulsivity and greater severity on scales that assess the core symptoms of disordered eating behavior and body dissatisfaction.

**Disclosure:**

No significant relationships.

